# Efficacy of radiomics model based on the concept of gross tumor volume and clinical target volume in predicting occult lymph node metastasis in non-small cell lung cancer

**DOI:** 10.3389/fonc.2023.1096364

**Published:** 2023-05-24

**Authors:** Chao Zeng, Wei Zhang, Meiyue Liu, Jianping Liu, Qiangxin Zheng, Jianing Li, Zhiwu Wang, Guogui Sun

**Affiliations:** ^1^ Hebei Key Laboratory of Medical-industrial Integration Precision Medicine, Clinical Medicine College, Affiliated Hospital, North China University of Science and Technology, Tangshan, Hebei, China; ^2^ Department of Radiotherapy, Yantai Yuhuangding Hospital, The Affiliated Hospital of Qingdao University, Yantai, Shandong, China; ^3^ Department of Chemoradiation, Tangshan People’s Hospital, Tangshan, Hebei, China

**Keywords:** radiomics, occult lymph node metastasis, non-small cell lung cancer, chest-enhanced CT, prediction model

## Abstract

**Objective:**

This study aimed to establish a predictive model for occult lymph node metastasis (LNM) in patients with clinical stage I-A non-small cell lung cancer (NSCLC) based on contrast-enhanced CT.

**Methods:**

A total of 598 patients with stage I–IIA NSCLC from different hospitals were randomized into the training and validation group. The “Radiomics” tool kit of AccuContour software was employed to extract the radiomics features of GTV and CTV from chest-enhanced CT arterial phase pictures. Then, the least absolute shrinkage and selection operator (LASSO) regression analysis was applied to reduce the number of variables and develop GTV, CTV, and GTV+CTV models for predicting occult lymph node metastasis (LNM).

**Results:**

Eight optimal radiomics features related to occult LNM were finally identified. The receiver operating characteristic (ROC) curves of the three models showed good predictive effects. The area under the curve (AUC) value of GTV, CTV, and GTV+CTV model in the training group was 0.845, 0.843, and 0.869, respectively. Similarly, the corresponding AUC values in the validation group were 0.821, 0.812, and 0.906. The combined GTV+CTV model exhibited a better predictive performance in the training and validation group by the Delong test (*p*<0.05). Moreover, the decision curve showed that the combined GTV+CTV predictive model was superior to the GTV or CTV model.

**Conclusion:**

The radiomics prediction models based on GTV and CTV can predict occult LNM in patients with clinical stage I–IIA NSCLC preoperatively, and the combined GTV+CTV model is the optimal strategy for clinical application.

## 1 Introduction

Lung cancer has been identified as the leading cause of cancer-related death worldwide, accounting for 18.0% of all cancer deaths. Non-small cell lung cancer (NSCLC) accounts for 80%–85% of all lung cancer cases (1). Low-dose computed tomography (CT) screening programs can identify the majority of patients with early-stage NSCLC. Currently, surgery combined with systemic therapy is dominant in the treatment of early resectable NSCLC (2, 3), and anatomical lobectomy and lung mediastinal lymph node cleaning are recommended. A larger range of lymph node cleaning can reduce the risk of lymph node metastasis, but physical trauma to the patient is also greatly increased, such as unnecessary normal tissue damage, pneumothorax, lymph node leakage, prolonged hospital stay, and other risks (4). Incidence of lymph node metastases (LNMs) in early lung cancer cases could reach 10% (5), but in some cases, the LNMs are occult. Consequently, if preoperative imaging can reliably identify negative lymph nodes, these patients can avoid lymph node dissection and lower surgical risks or receive stereotactic radiotherapy and other local treatments as alternative approaches. At present, CT, magnetic resonance imaging (MRI), and positron emission computed tomography (PET-CT) are commonly used clinically for the non-invasive evaluation of lymph nodes, among which PET-CT is the best method to evaluate lymph node status (6–8). However, currently, there is no imaging diagnostic method with high accuracy for occult LNM (short diameter ≤1 cm) (9).

In recent years, the wave of precision medical radiomics drew widespread attention (10–12). Radiomics refers to the high-throughput extraction and analysis of a large number of advanced and quantitative imaging features from medical imaging such as CT, PET, or MRI (13–15). The features in radiomics provide information about tumor phenotype and microenvironment that is far richer than that obtained visually. Accumulating studies have attempted to predict the status of lymph node status using the radiomics features of the primary lesions in lung cancer (16). However, these features were mostly derived from (primary tumor volume) GTV, and the features of the microinvasion area around the tumor, termed as clinical target volume (CTV), were ignored. Herein, the authors extracted radiomics indexes of GTV and CTV from CT images using the radiomics tool. Based on GTV, CTV, and GTV+CTV indexes, we developed three predictive model for occult lymph node metastasis (LNM) in stage I–IIA NSCLC and validate radiomics prediction models to predict occult LNM in NSCLC with clinical stage I–IIA.

## 2 Patients and methods

### 2.1 Patients

In this retrospective study, all data have been de-identified, and no experiments were performed on patients. Therefore, the Institutional Review Board of Tangshan People’s Hospital allowed waiver of informed consent. All methods were carried out in accordance with relevant guidelines and regulations. All experimental protocols were approved by the Institutional Review Board of Tangshan People’s Hospital. A total of 401 patients with lung cancer undergoing radical surgery in Tangshan People’s Hospital from September 2017 to December 2019 and 197 patients in Yantai Yuhuangding Hospital from 2016 to 2020 were included. The age range was 29–83 years old, with a median age of 62 years old, including 273 men and 325 women. The status of lymph node was classified as lymph node metastasis or no lymph node metastasis based on postoperative pathological results as shown in **Table 1**. Inclusion criteria were the following: [I] patients with preoperative clinical stage I–IIA (UICC eighth edition TNM staging) underwent radical surgery and systemic lymph node dissection; [II] postoperative pathology was NSCLC with or without lymph node metastasis; [III] all patients received contrast-enhanced chest computed tomography (contrast-enhanced computed tomography) with the same type of CT equipment 1 month before the operation; and [IV] short diameter of Lymph nodes were <1cm on CT images. Exclusion criteria were as follows: [I] patients had received neoadjuvant therapy before surgery, [II] had other malignant tumors, and [III] had distant metastasis.

### 2.2 CT Imaging acquisition and preprocessing

**Figure 1:**
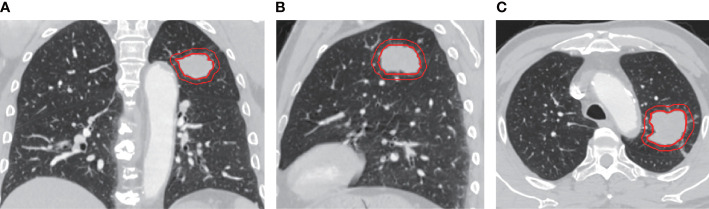
Representative images of Chest enhanced CT illustrating gross tumor volume (GTV) and clinical target volume (CTV) with 5mm expansion from the coronal **(A)**, sagittal **(B)**, and axial **(C)** view. The inner and outer red circle represents GTV and CTV, respectively.

All patients were in supine position and scanned from the thoracic entrance to the base of the diaphragm. Scanning setting was as follows: scanning thickness, 5.0 mm; tube voltage, 120 kVP; and tube current, 80–300 mAs. All images were displayed in the standard lung (width, 1,200 HU; level, 600 HU) and mediastinal (width, 350 HU; level, 40 HU) Windows settings. An iodinated contrast agent of 1.5–2 ml/kg was injected through the cubital vein at a rate of 3 ml/s, and the venous phase scan was delayed by 90 s. All images were saved to a hard drive in the DICOM (Digital Imaging and Medical Communication) format. Images were then imported into the Accu Contour software from Manteia (version 3.1), where GTV was manually delineated by radiologists with more than 5 years of experience who were not aware of any clinical or pathological information. Tumor GTV includes tumor parenchyma, interstitial blood vessels, and vacuoles within nodules, excluding normal lung tissue. CTV refers to the area around the GTV with an external expansion of 5 mm, excluding the GTV area (**Figure 1**). The manual repair was performed if the outer expansion came into contact with the chest wall, diaphragm, main trachea, great blood vessels, or heart. 

### 2.3 Radiomics feature extraction

The “Radiomics” tool kit in AccuContour software was used to extract the radiomics features of GTV and CTV delineated. It includes 14 shape features and 252 first-order statistics), 336 gray level co-occurrence matrix (GLCM) features, 70 neighborhood gray level difference matrix (Ngtdm) features, 180 running length matrix (RLM) features, 224 gray level region matrix (GLSZM) features, and 238 gray level dependence matrix (GLDM) features, a total of 1,314 radiomics features.

### 2.4 Model construction

The cases were divided into a training group and validation group with a ratio of 2:1. Models were constructed in the training group and were validated in the external validation group. A total of three prediction models were built by incorporating different feature types, namely, the GTV radiomics model, the CTV radiomics model, and the GTV+CTV hybrid radiomics model.

### 2.5 Analysis of data

Statistical analysis was performed using R software (R-4.1.3patched) A total of 1,314 extracted radiomics features were first screened using the least absolute shrinkage and selection operator (LASSO) logistic regression algorithm to select features with non-zero coefficients. The screened features were used to build logistic regression models, and the stepwise regression method (stepAIC) was employed to test and remove the non-significant variables. A radiomics nomogram was constructed to quantify the individual probability of OLNM for the significant variables, and the relationship between the predicted and ideal values was assessed using calibration curves. The area under the curve (AUC) was calculated by receiver operating characteristic (ROC).

## 3 Results

### 3.1 Clinical characteristics and univariate analysis

A total of 598 enrolled patients were randomized into the training (*N*=401) and validation (*N*=197) cohorts. As can be seen from **Table 1**, no significant difference in clinical characteristics (age, gender, T stage, tumor site, and pathological classification) was found between lymph node metastasis positive and negative subgroups, whether in training or validation cohort. Therefore, patients in two subgroups were comparable for all clinical factors whether in training or validation cohort.

**Table 1:** Clinical characteristics of patients with lung cancer.

	Training group			Validation group		
	LNM (+)	LNM (−)	*p*-value	LNM (+)	LNM (−)	*p*-value
Age	60.03 ± 9.80	61.35 ± 8.01	0.248	59.67 ± 9.50	60.22 ± 9.36	0.718
Gender						
Male	28	149	0.644	25	68	0.916
Female	39	185		27	77	
T stage						
T1	50	284	0.000	27	130	0.071
T2	17	50		25	15	
Tumor site						
Upper right	19	105	0.381	19	47	0.056
Right middle	15	59		6	28	
Lower right	5	21		1	9	
Upper left	18	82		9	38	
lower left	10	67		17	23	
PC						
AdC	53	290	0.281	43	127	0.350
SCC	14	44		9	18	

LNM, lymph node metastasis; PC, pathological classification; ADC, adenocarcinoma; SCC, squamous cell carcinomas.

### 3.2 Radiomics feature extraction and screening

After identifying the region of interest (ROI) for the tumor volume, the CTV was created through Boolean logic extrapolation. The “Radiomics” plugin in AccuContour software (https://www.radiomics.io/PyRadiomics.html) was then utilized to extract image histology features from each ROI. The plugin automatically extracted 1,314 radiomics features from GTV and CTV images, and a hybrid model combining the GTV and CTV features was also developed. Finally, the most effective variables were selected from the training set of the three models by employing 10-fold cross-validation using LASSO regression (**Table 2**).

**Table 2:** Parameters applied in the constructed predictive models.

Features	Name	Feature type	Image type
Feature92	Dependence variance	GLDM	Original
Feature131	Correlation	GLCM	Wavelet-LLH
Feature220	Cluster prominence	GLCM	Wavelet-LHL
Feature371	Dependence variance	GLDM	Wavelet-LHH
Feature902	Run entropy	GLRLM	Square
Feature1132	90th percentile	First order	Exponential
Feature1205	Dependence entropy	GLDM	Exponential
Feature1306	Large dependence high gray level emphasis	GLDM	Gradient

GLCM, gray level co-occurrence matrix; GLDM, gray level dependence matrix; GLRLM, gray level travel length matrix.

### 3.3 Model construction and model evaluation

**Figure 2:**
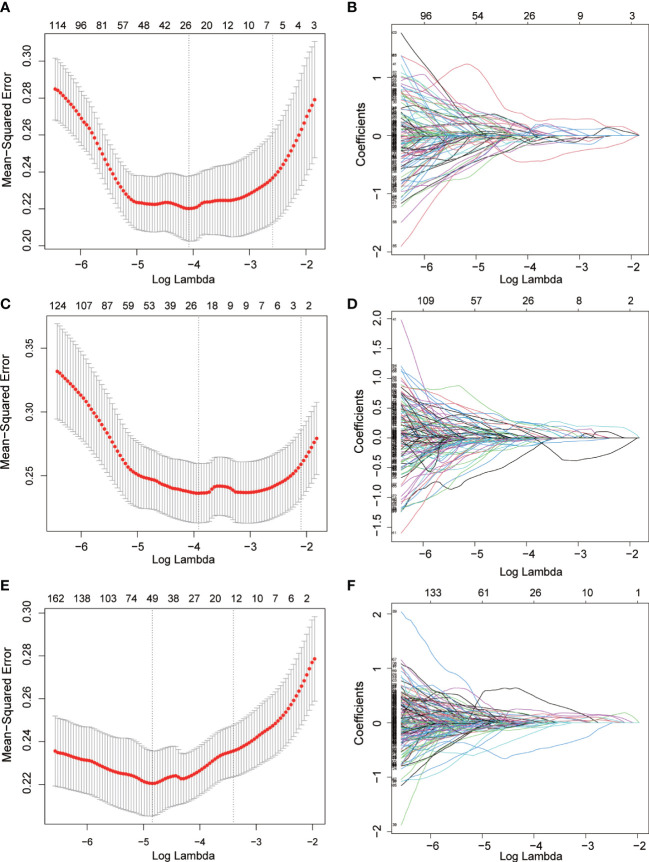
Radiomics features screened using the LASSO regression model. **(A, C, E)** the mean squared error of radiomics features displayed by the Lasso regression analysis in GTV, CTV and GTV+CTV model. Two vertical lines represented the lambda values when number of variables decreased to two lowest levels. **(B, D, F)** The trajectory of each radiomics feature in GTV, CTV and GTV+CTV model. The horizontal axis represents the log lambda of each radiomics, and the vertical axis represents the coefficient of each radiomics.

**Figure 3:**
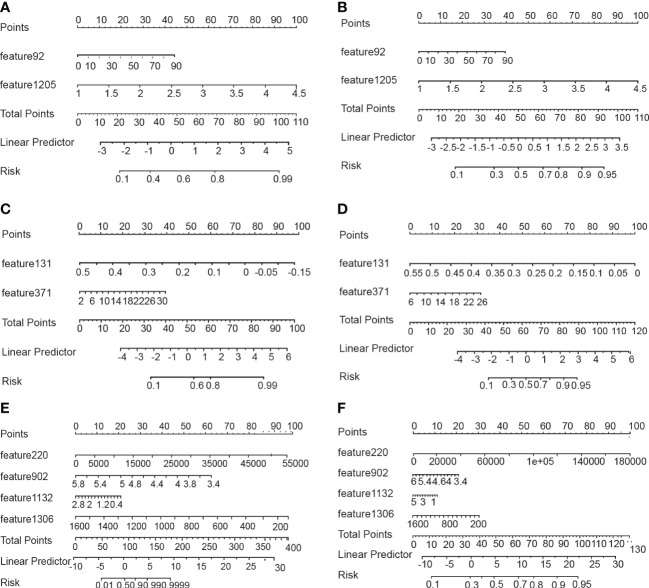
Nomograms for predicting lymph node metastasis. **(A, B)** Nomograms using the GTV algorithm in the training and validatiaon cohort. **(C, D)** Nomograms using the CTV algorithm in the training and validatiaon cohort. **(E, F)** Nomograms using the GTV+CTV algorithm in the training and validatiaon cohort.

**Figure 4:**
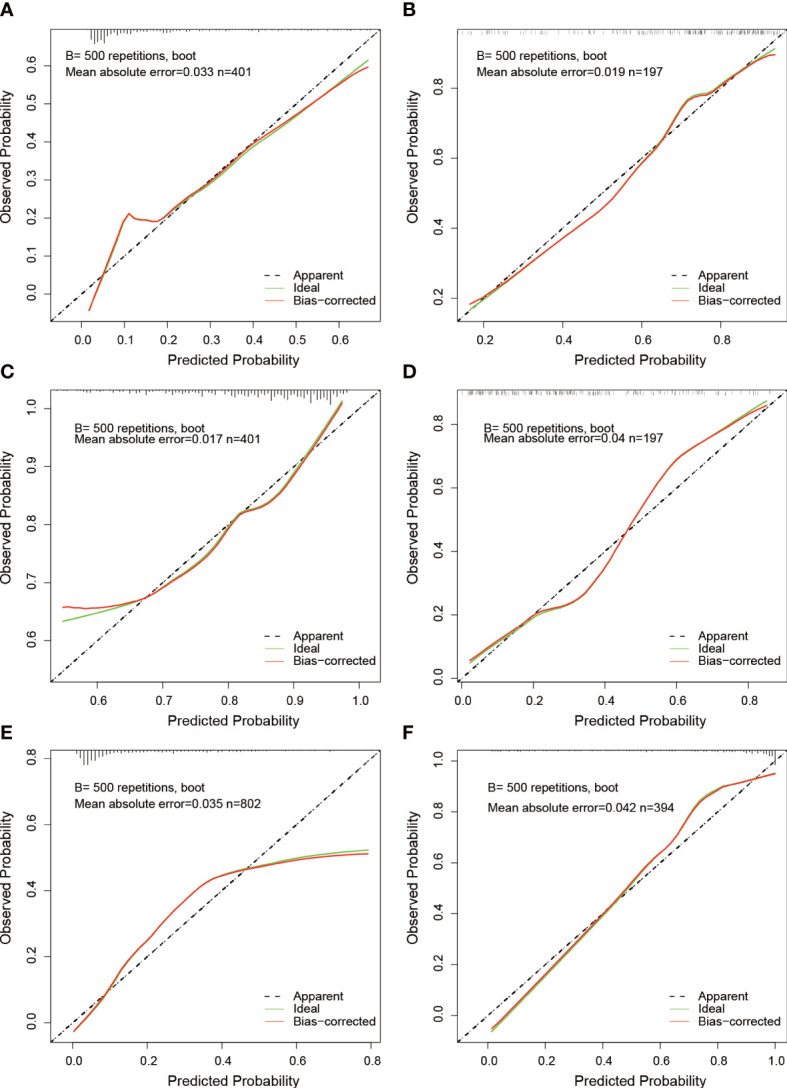
Calibration curves of nomograms. **(A, B)** Calibration curves for GTV predictive model at the training and validation cohort. **(C, D)** Calibration curves for CTV predictive model at the training and validation cohort. **(E, F)** Calibration curves for GTV+CTV predictive model at the training and validation cohort.

To avoid overfitting of the model, LASSO regression was first employed to reduce the number of variables (features). As can be seen from **Figure 2**, 4, 2, and 11 features in GTV, CTV, and combined model, respectively, were filtered out for subsequent multi-Cox regression, respectively. Through multi-Cox regression analysis, the variable number of GTV, CTV, and combined GTV+CTV model was further reduced. Ultimately, a total of eight features, including feature 92, feature 131, feature 220, feature 371, feature 902, feature 1,132, feature 1,205, and feature 1,306, were identified (**Table 2**). Moreover, six nomogram plots were drawn to present the built models (**Figure 3**). On the left side of the nomogram plots, risk factor variables, the overall score, and the likelihood of predicted lymph node metastasis were displayed from top to bottom. The nomogram method involved finding the corresponding scores according to the assigned values of each risk factor variable, adding the scores to obtain the total score, and then determining the risk of lymph node metastasis that corresponds to the total score. Several groups of multi-factor logistic regression models were built using different features for GTV, CTV, and GTV+CTV. The models were subsequently tested for their effectiveness using an external validation group. Calibration curves were plotted to evaluate the agreement between the predicted incidence and actual incidence in the training group, external validation group, and mixed group models, respectively (**Figure 4**).

### 3.4 Comparison of models

**Figure 5:**
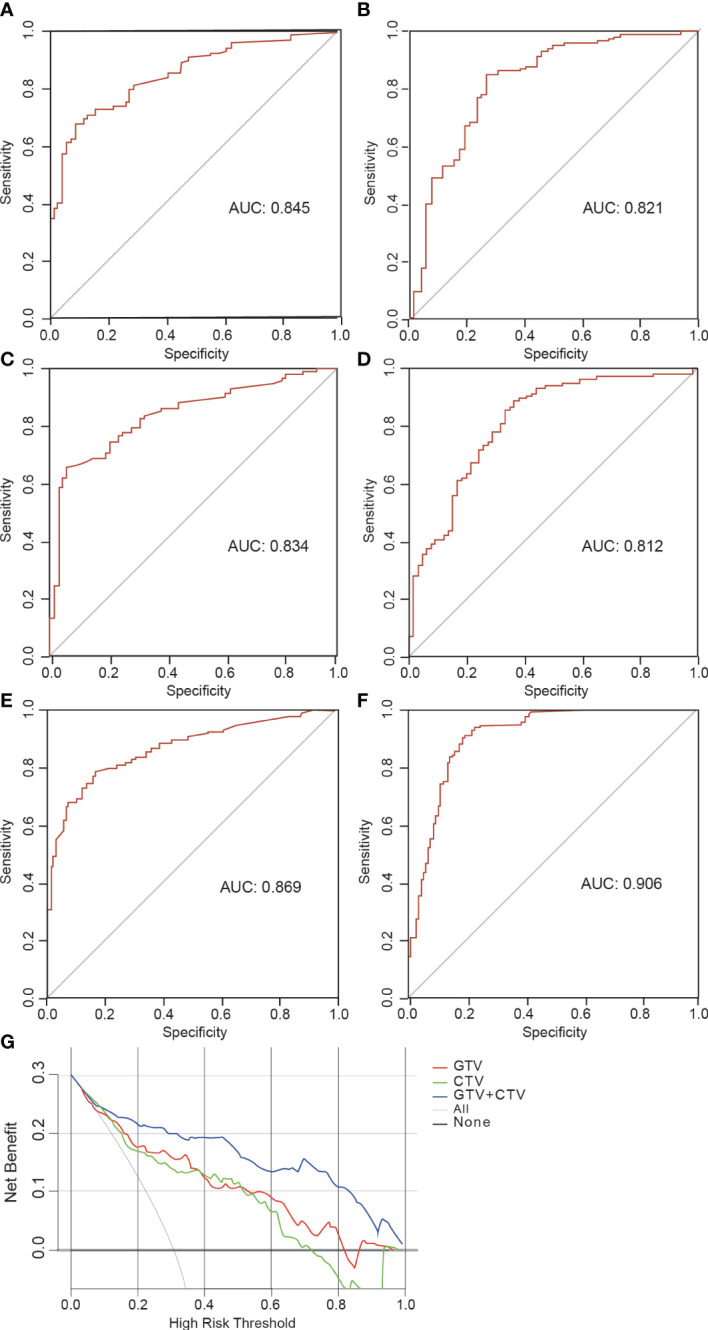
Receiver operating characteristic (ROC) curve and decision curve. **(A, B)** The ROC curves illustrating AUC values of GTV model at the training and validation cohort. **(C, D)** The ROC curves illustrating AUC values of CTV model at the training and validation cohort. **(E, F)** The ROC curves illustrating AUC values of GTV+CTV model at the training and validation cohort. **(G)** Decision curve analysis for GTV, CTV and GTV+CTV models.

The AUCs of the GTV model based on the features from GTV was 0.845 (95% CI, 0.801–0.888) in the training and 0.821 (95% CI, 0.749–0.894) in the validation group, respectively. The AUC values for the CTV model were 0.834 in the training (95% CI, 0.787–0.834) and 0.812 (95% CI, 0.780–0.912) in the validation group, respectively. The AUC values for the GTV+CTV hybrid model were 0.869 (95% CI, 0.842–0.896) and 0.906 (95% CI, 0.757–0.953) in the training and validation group, respectively (**Table 3**). The higher the AUC value, the better the prediction effect of the model, and the ROC curve of each model is shown in **Figure 5**. The AUC values of the CTV model compared with the GTV model in the external validation group did not show any advantage (Delong’s test p-value both GTV and CTV groups were all >0.05, 0.735 in the training group and 0.855 in the validation group, which was not statistically significant). The AUC values of the hybrid GTV+CTV model were higher than those of the GTV and CTV validation group models (the AUC of the hybrid model was 0.869 in the training group and 0.906 in the validation group). The Decision curve is used to check the net benefit of each model, among which the hybrid model has the best net benefit (**Figure 5**). The p-values of the Delong test between the training and validation groups hybrid model and the remaining two groups were <0.05, which was statistically significant (the p-value of the Delong test between the hybrid and GTV training groups was 0.01, the p-value of the Delong test between the hybrid and GTV training groups was 0.02, the p-value of the Delong test between the hybrid and GTV validation groups was 0.04, and the p-value of the Delong test between the hybrid and CTV validation groups was 0.01).

**Table 3:** Parameters of ROC curves in GTV, CTV, and GTV+CTV model.

	GTV model	CTV model	GTV+CTV model
Training group			
Sensitivity	0.673	0.650	0.783
Specificity	0.912	0.940	0.838
Youden index	0.585	0.590	0.621
AUC (95%CI)	0.845 (0.801–0.888)	0.834 (0.787–0.834)	0.869 (0.842–0.896)
Validation group			
Sensitivity	0.731	0.885	0.902
Specificity	0.848	0.636	0.814
Youden index	0.579	0.521	0.716
AUC (95%CI)	0.821 (0.749–0.894)	0.812 (0.780–0.912)	0.906 (0.870–0.942)

## 4 Discussion

In this study, we constructed three models to predict occult lymph node metastasis in NSCLC based on radiomics features. In particular, the hybrid GTV+CTV model exhibited the best performance in predicting the status of lymph node (AUC of 0.869 in the training group and AUC of 0.906 in the validation group).

In the prediction of occult LNM in lung cancer, two types of radiomics features, GLCM (25%) and GLDM (50%), were found to be the most prevalent. Compared with clinical features, the radiomics features demonstrated better predictive efficacy. The continued predictive value of these features for other types of cancer requires further confirmation. The predictive ability of the GTV or CTV model was not satisfactory, probably due to the relatively small sample size in this study (only 401 cases were included in our hospital, and an additional 197 cases came from elsewhere). However, the mixed training model and validation model by combining GTV and CTV demonstrated improved predictive power, probably due to the larger sample size and increased exposure rate of effective features. Therefore, radiomics studies may achieve more accurate results with larger sample sizes. A multicenter study design employed in this study aimed to eliminate any potential single-center bias. Many studies predicting lymph node metastasis in a variety of fields, including lung cancer, bile duct cancer, gastric cancer, and colorectal cancer, facilitated the development of radiomics in recent years (10, 16–18). The majority of studies concentrated on features extracted from the primary tumor area in images, ignoring the role of the environment around the primary tumor, which we called CTV in this study (19, 20). According to the definition of CTV in radiotherapy, the CTV is GTV and the area around GTV, which is suspected to be microscopic extension of the tumor. Although CTV is invisible, it may contain some microscopic information, which can be reflected by the features of radiomics, and these features can predict LNM (11, 21). In fact, this study did find that the joint GTV and CTV model had stronger predictive power than the GTV model.

Some studies reported that clinical features such as tumor size, density, morphological features, and serum CEA were highly correlated with lymph node metastasis in lung cancer (22, 23), and some studies confirmed that radiomics features combined with clinical features could predict lymph node metastasis (10, 24, 25). There are two well-known models that were constructed to predict the probability of lymph node metastasis in NSCLC patients. The Help with the assessment of adenopathy in lung cancer (HAL) model was used to predict pN2/N3 in patients with NSCLC, and the Help with Oncologic Mediastinal Evaluation for Radiation (HOMER) model can estimate the probability of N0+N1 in patients with NSCLC (26–28). Unlike our model, which was used to predict the presence or absence of lymph node metastasis, these two models were used to predict N0+N1 and N2+N3 in patients with NSCLC. Our model and these two models have different clinical uses, so the two types of models cannot replace each other.

Fluorine-18-fludeoxyglucose (18F-FDG) positron emission tomography/computed tomography (PET/CT) can non-invasively detect the metabolic activity of the disease. Compared with traditional CT images, PET images contain more metabolism information, which reflects the aggressiveness of the tumor. The models based on PET-CT imaging performed well in the prediction of occult LNM, with AUC values ranging from 0.769 to 0.881 (29, 30). Surprisingly, similar models based on traditional CT imaging could achieve comparable predictive efficacy. For example, the model of Zhang et al. based on CT and clinical features could predict the lymph node status of stage I–II NSCLC, with the AUC value of 0.88 (31). However, Das et al. developed a model to predict the lymph node status of cT1N0M0 lung adenocarcinoma, either alone or in combination with clinical features. The external validation group yielded an AUC of 0.79 (95% CI, 0.66–0.93) (32). In our study, the combined GTV+CTV model based on traditional CT imaging also exhibited a comparable predictive ability with an AUC value of 0.845. In view of acceptable predictive ability and lower price of CT imaging, the combined GTV+CTV predictive model based on traditional CT imaging is worthy of clinical application.

The small number of cases and inclusion of clinical features in this study, however, were thought to be related to the clinical features’ poor predictive performance, so only radiomics features were included. The predictive advantage of the radiomics model was also significant, and in comparison to the radiomics model based on the bulk tumor volume, the combined model including the CTV had a higher predictive efficacy, which was thought to be related to the increased patient population.

This study solely focused on NSCLC, where the tumor bulk volume under enhanced CT was explicitly outlined. To predict occult lymph node metastasis, patients with positive lymph nodes in their imaging report were excluded. Complete data, including the CT report and postoperative pathological reports, were collected, and an external test group was added to investigate the influencing factors thoroughly. Three different models were generated by incorporating various traits. The integrated radiomics model of tumor bulk volume and CTV displayed superior predictive efficacy for occult lymph node metastasis in NSCLC, which was better reflected through comparing the three models.

This study has several limitations: first, despite being a multicenter study, the number of cases with positive lymph nodes was much smaller than the number without lymph node metastasis. Second, this study only explored the relationship between radiomics features and LNM and did not explore the relationship between other factors such as clinical information and LNM. We will further refine our study in the future.

## 5 Conclusion

Overall, there is currently a shortage of affordable, convenient, and safe methods for predicting LNM in patients with early-stage lung cancer. The radiomics prediction models based on GTV and CTV can predict occult LNM in patients with clinical I–IIA stage NSCLC preoperatively, and the combined GTV+CTV model is optimal strategy for clinical application.

## Data availability statement

The original contributions presented in the study are included in the article/supplementary material. Further inquiries can be directed to the corresponding authors.

## Author contributions

GS, ZW, and WZ contributed to the conception and design of the study. ML, JNL, and QZ organized the database. Statistical analysis was performed by JPL and ZC. ZC wrote the first draft of the manuscript. ZC and WZ wrote parts of the manuscript. All authors contributed to the article and approved the submitted version.
